# Specific tracking of N-terminal clipping on histone H3 in *Tetrahymena* enabled by a custom branched-peptide antibody

**DOI:** 10.1007/s42995-025-00351-4

**Published:** 2026-01-14

**Authors:** Fan Wei, Bo Pan, Xiangning Han, Saleh A. Al-Farraj, Jianxin Sui, Shan Gao

**Affiliations:** 1 MOE Key Laboratory of Evolution and Marine Biodiversity and Institute of Evolution and Marine Biodiversity, Ocean University of China, Qingdao, 266003 China; 2 Laboratory for Marine Biology and Biotechnology, Qingdao Marine Science and Technology Center, Qingdao, 266237 China; 3 Present Address: Department of Pharmacology and Cancer Biology, Duke University School of Medicine, Durham, NC 27710 USA; 4 State Key Laboratory of Marine Food Processing and Safety Control, College of Food Science and Engineering, Ocean University of China, Qingdao, 266404 China; 5 Present Address: Department of Chemical Biology, School of Life Sciences, Southern University of Science and Technology, Shenzhen, 518055 China; 6 Department Zoology, College of Science, King Saud University, Riyadh, 11451 Saudi Arabia

**Keywords:** Branched-peptide antibody, Histone H3 clipping, Histone post-translational modification, In situ detection, *Tetrahymena thermophila*

## Abstract

Histone H3 clipping, a unique but evolutionarily conserved post-translational modification that irreversibly removes the N-terminal tail of H3, has been reported across diverse eukaryotic lineages. In *Tetrahymena thermophila*, a ciliate with nuclear dimorphism, H3 clipping is a *bona fide* proteolytic event generating H3^F^ (H3-Fast) by removing the first six N-terminal amino acids, specifically in the transcriptionally silent micronucleus (MIC). However, the detection of H3^F^ remains technically demanding, time-consuming, and lacks spatio-temporal resolution. To overcome this, a 2 × branched peptide antigen was developed to generate a high-specificity antibody that exclusively recognizes H3^F^, effectively distinguishing it from full-length H3 and other truncation variants. This antibody eliminated the need for labor-intensive MIC isolation and histone extraction, enabling rapid, small-scale detection directly from whole-cell lysates. Using this antibody, dynamic subcellular localization of H3^F^ was investigated through different cell stages, revealing its persistence during vegetation, starvation and early conjugation. However, H3^F^ disappeared concurrently with macronuclear anlage formation, supporting the notation that removal of H3^F^ is a prerequisite for the new macronucleus development. Comparative analyses further revealed that H3 Ser10 phosphorylation, though previously used as an alternative H3^F^ marker, actually occurs strictly after clipping, refining the temporal hierarchy of these two chromatin events. This work provides the first in situ, high-resolution method to track endogenous H3 clipping, providing both a technical platform and new biological insight into the developmental regulation of proteolytic histone modifications.

**Supplementary Information:**

The online version contains supplementary material available at 10.1007/s42995-025-00351-4.

## Introduction

In eukaryotes, genomic DNA is compacted into chromatin, whose fundamental unit is the nucleosome. Each nucleosome consists of approximately 147 base pairs of DNA wrapped around an octamer of core histone proteins, including two copies each of H2A, H2B, H3, and H4 (Villar-Garea and Imhof 2006). Among these, histone H3 plays a central role in chromatin structure and regulation, particularly through its N-terminal tail (Luger et al. 1997; Strahl and Allis 2000). This tail, rich in positively charged residues such as lysine and arginine, interacts with the negatively charged DNA backbone, stabilizing nucleosome structure and contributing to higher-order chromatin organization (Kornberg and Lorch 1999; Luger et al. 1997). It is also a hotspot for post-translational modifications (PTMs), including acetylation, methylation, phosphorylation, and ubiquitination (Brownell et al. 1996; Gao et al. 2013; Hendzel et al. 1997; Ng et al. 2003; Wang et al. 2006). These modifications mediate the dynamic regulation of gene expression, DNA repair, and chromatin accessibility (Bannister and Kouzarides 2011; Hendzel et al. 1997; Kouzarides 2007; Ng et al. 2003; Papazyan et al. 2014; Wang et al. 2006; Wei et al. 1999).

**Fig. 1:**
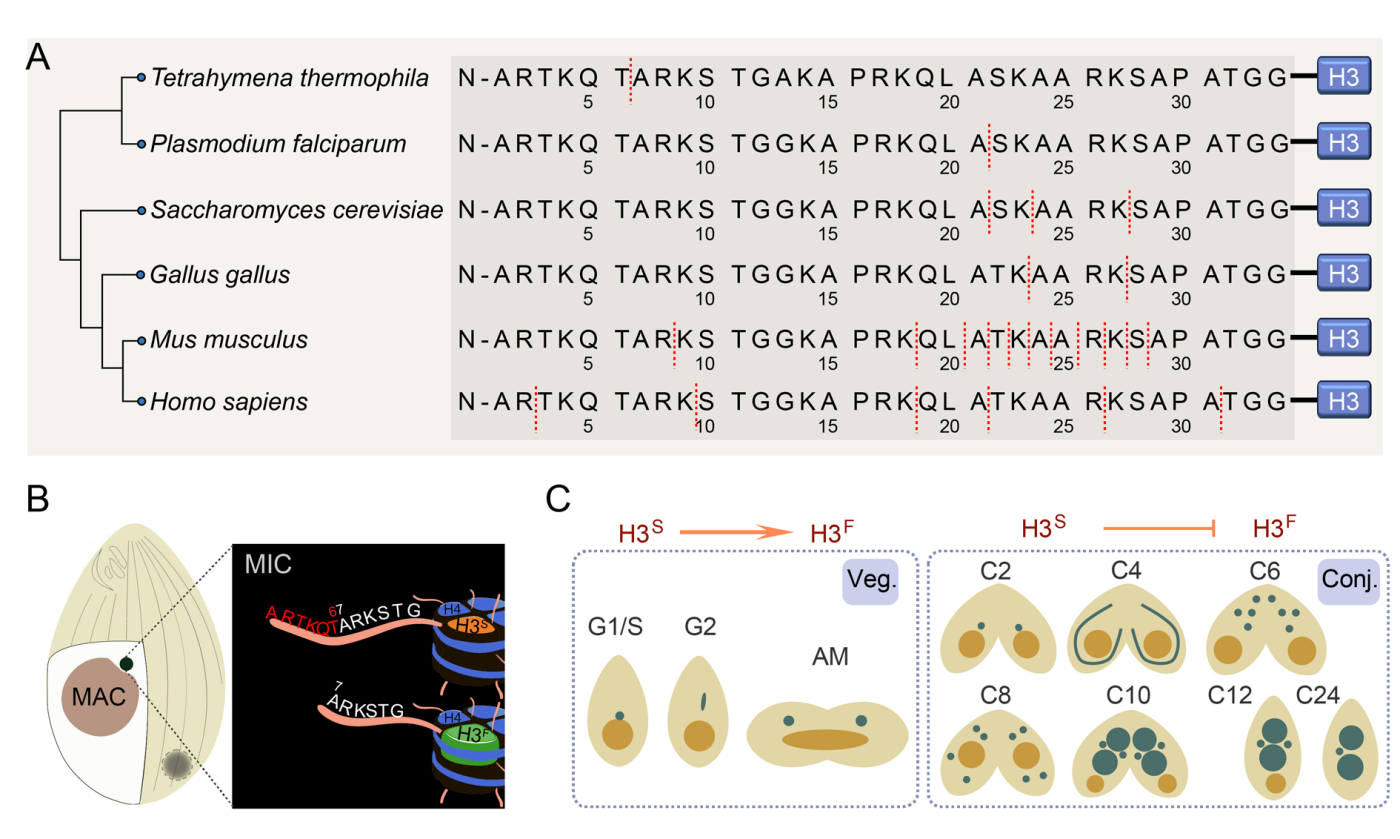
Characterization of H3 clipping in *Tetrahymena thermophila*. **A** Schematic representation of histone H3 N-terminal tail sequences across diverse eukaryotes. Red dashed lines indicate reported cleavage sites. The H3 cleavage sites are predominantly clustered around the 21^st^ amino acid across most eukaryotes. In contrast, the cleavage site in *Tetrahymena* represents a rare N-terminal truncation occurring significantly closer to the N-terminus. **B** *Tetrahymena* cell containing a germline micronucleus (MIC) and a somatic macronucleus (MAC). H3 clipping occurs exclusively in MIC by removing the first six amino acids (red letters) on H3^S^ to form H3^F^. **C** H3 clipping occurs during the vegetative stage (veg.) of *Tetrahymena*, but not conjugation stage (conj.)

Beyond canonical PTMs, the N-terminal tail of histone H3 undergoes proteolytic clipping, an irreversible processing that removes a defined segment from the amino terminus (Dhaenens 2021; Dhaenens et al. 2015; Gunjan et al. 2006; Howe and Gamble 2015; Yi and Kim 2018; Zhou et al. 2014). For example, in mouse or human embryonic stem cells, H3 N-terminal tails are predominantly truncated at Ala21 and Thr22 (Duncan et al. 2008), whereas in yeast, cleavage occurs between Lys23 and Ala24 (Xue et al. 2014), underscoring the substantial diversity of H3 clipping sites across species (Fig. 1A) (Wei et al. 2022). This clipping event disrupts DNA-histone interactions, eliminates PTM sites, and may reshape the chromatin landscape. In mammalian systems, H3 clipping has been attributed to specific proteases such as cathepsin L, with implications for transcriptional reprogramming and cell development (Duncan et al. 2008). However, the physiological roles, timing, and regulation of H3 clipping remain poorly understood in most systems due to its elusive characteristics and the lack of robust detection tools.

Conventional approaches for the detection of the H3 clipping product include: (1) mass spectrometry (MS): to identify N-terminal truncation sites via peptide mapping; (2) pulse-chase labeling: to monitor the synthesis and processing of histones over time; (3) Western blot (WB): to distinguish full-length and truncated H3 using pan-H3 antibodies; and (4) immunofluorescence (IF): to visualize the spatial distribution of truncated H3 within nuclei. Among these, MS and pulse-chase are antibody-independent but often require large amounts of input materials and involve technically demanding procedures. WB and IF, on the other hand, rely entirely on the availability and specificity of antibodies to distinguish different H3 forms. However, they suffer from low spatial resolution; in particular, general H3 antibodies are incapable of distinguishing truncated and full-length H3 at the single-cell level by IF staining. Overall, the inability of these approaches to directly visualize endogenous H3 clipping events in situ limits their application in dynamic and developmental contexts. These limitations underscore the need for novel tools capable of high-resolution, context-dependent tracking of H3 clipping products.

*Tetrahymena thermophila* (referred to as *Tetrahymena* hereafter) is a well-known model organism for investigating chromatin biology and histone dynamics (Brownell et al. 1996; Chalker et al. 2013; Hao et al. 2024; Papazyan et al. 2014; Wang et al. 2025; Ye et al. 2025; Zhang et al. 2025). Like other ciliates, it features nuclear dualism, maintaining two functionally distinct nuclei within a single cell: a transcriptionally active macronucleus (MAC) that governs somatic functions, and a transcriptionally silent micronucleus (MIC) that serves as the germline (Fig. 1B) (Jin et al. 2023; Karrer 2012; Lyu et al. 2024). During sexual reproduction (conjugation), the MAC degrades and the new MAC and MIC both develop from a zygotic nucleus derived from parental MICs (Fig. 1C) (Orias et al. 2011). This well-defined nuclear differentiation program, along with distinct chromatin landscapes, provides an exceptional in vivo context to study nucleus-specific histone modifications.

*Tetrahymena* is one of the first systems in which histone H3 clipping was discovered. By utilizing two-dimensional acid-urea (2D-AU) gel electrophoresis, a faster-migrating H3 variant, termed H3^F^ (H3-Fast) specifically in MICs of vegetatively growing *Tetrahymena* (Allis et al. 1980, 1979). Amino acid sequencing revealed that H3^F^ arises from the removal of the first six N-terminal residues from its precursor H3^S^ (H3-Slow), which is similar to MAC H3 if not identical (Fig. 1A, B). Pulse-chase labeling with [^3^H]-labeled lysine further demonstrated that H3 clipping in *Tetrahymena* is strictly restricted to the MIC and occurs exclusively during vegetative growth but not starvation or conjugation (Fig. 1C) (Allis and Wiggins 1984b). Notably, some H3^F^ persists in the early stages of conjugation, while the exact time point of its disappearance has yet to be determined. The absence of H3 clipping during conjugation has been hypothesized to facilitate the development of the new MAC that is free of H3^F^ (Allis and Wiggins 1984b), suggesting a potential biological necessity for terminating this modification during nuclear differentiation. Moreover, when conjugating progenies were transferred back to growth medium, H3 clipping reappeared after one round of cell division, highlighting a strong link between clipping and cell cycle (Allis and Wiggins 1984b). WB using H3 antibodies also revealed distinct bands corresponding to H3^S^ and H3^F^ (Papazyan et al. 2014). Notably, MS further characterized this proteolytic process and indicated that PTMs were differentially enriched: mitosis-specific mark H3S10ph and chromatin condensation-associated mark H3K23me3 were exclusively detected on H3^F^ (Papazyan et al. 2014). Moreover, the mutagenesis studies demonstrated that substituting serine 10 with alanine (H3S10A) did not eliminate H3 clipping, as the H3^F^ form could still be detected in such mutants (Wei et al. 1999). Together, these findings indicate that H3 clipping in *Tetrahymena* is a developmentally regulated, MIC-specific chromatin remodeling mechanism that likely coordinates nuclear identity along with cell cycle.

In *Tetrahymena*, truncated and full-length H3s differ by only six amino acids, making their separation and quantification more challenging than in other eukaryotes, where truncation typically removes about 20 amino acids (Fig. 1A) (Wei et al. 2022). Resolving these closely sized forms often requires extended gel electrophoresis. Furthermore, traditional approaches require labor-intensive MIC isolation and acid-based histone extraction (Allis et al. 1979; Papazyan et al. 2014). Although modification-specific antibodies (e.g., H3S10ph and H3K23me3) have been used to infer the presence of H3^F^, they may not reliably reflect its precise localization or abundance. Taken together, these observations highlight the urgent need for a robust method to directly detect endogenous H3^F^ and explore its subcellular localization during the cell cycle.

**Fig. 2:**
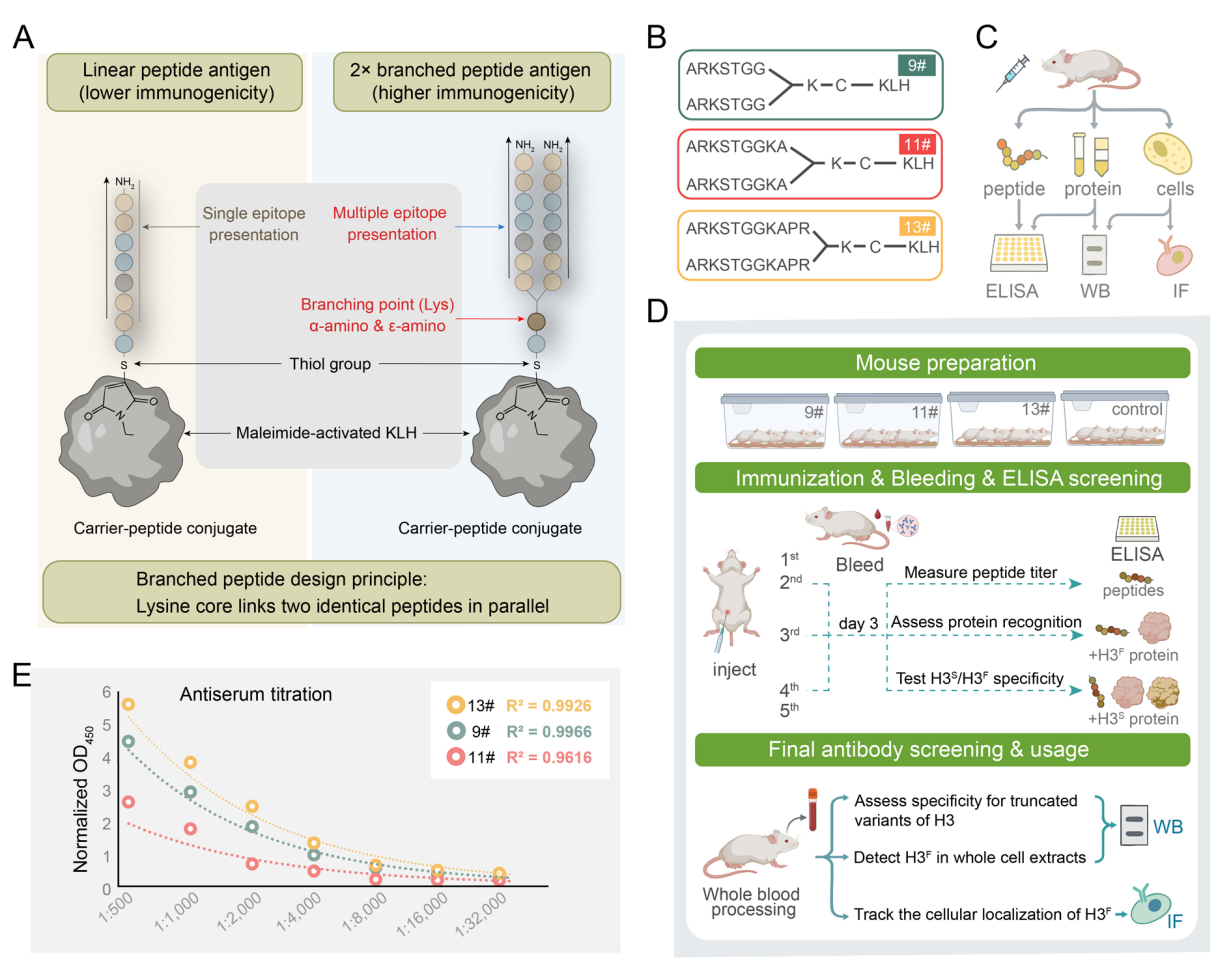
Strategic design of branched peptide antigens and immunization workflow for generating H3^F^-specific antibodies. **A** Schematic representation of linear and 2 × branched peptide-KLH conjugation. Branched peptide structure was designed by introducing a Lysine (K) residue at the C-terminus to serve as a branching point. The α-amino and ε-amino groups of the Lysine link two identical peptide sequences in parallel, facilitating multiple epitope presentation. Furthermore, the thiol group (-SH) of the Cysteine forms a thioether bond with the maleimide-activated KLH, ensuring that the N-terminal epitopes are fully exposed to the immune system for optimal recognition. **B** To mimic the exposed N-terminus of truncated H3^F^, three 2 × branched peptides of varying lengths (9#, 11#, and 13#) were synthesized for antibody generation. These peptides consist of nine, 11, and 13 amino acids, respectively, all starting from the 7^th^ position of the full-length H3 sequence and are conjugated to KLH via the C-terminal Cysteine residue. **C** Workflow for H3^F^ antibody generation. Mice were immunized with the antigen 9#, 11#, and 13#, and the resulting antisera (antibody) were evaluated by ELISA (using peptide or recombinant protein as antigens), by WB (with recombinant protein and cell lysates), and by IF (on fixed cells). **D** Detailed immunization and antibody screening timeline. Mouse preparation: four groups of mice (n = 5 per group) were established, including three experimental groups (9#, 11#, 13#) and one control group. Immunization and bleeding: a total of five injections were performed. Tail vein blood was collected three days post-immunization starting from the second injection for ELISA screening. Screening strategy: following the 2^nd^ injection, titers were measured against synthetic peptides. Post-3^rd^ injection, recognition of recombinant H3^F^ protein was assessed. Post-4^th^ injection, cross-reactivity between H3^S^ and H3^F^ was tested to ensure isoform specificity. After the final boost, whole blood was processed for comprehensive validation. Assess cross-reactivity with various truncated H3 variants by WB detection. Detect the recognition of H3^F^ in whole-cell extracts, also. Performing IF to track the cellular localization of H3^F^. **E** ELISA dilution curves of antisera from three immunized mice. Antisera were serially diluted from 1:500 to 1:32,000 to assess antigen-specific binding. Normalized OD_450_ values reflect antibody binding levels. Antisera from mouse 13# exhibited the highest binding activity to the peptide antigen

In this study, the N-terminal 2 × branched peptides of H3^F^ were designed to generate an H3^F^-specific antibody for the straightforward detection of H3^F^ (Fig. 2A, B). This antibody was able to selectively recognize H3^F^ without cross-reacting with H3^S^ or any other truncation variants. Using this antibody, a streamlined workflow was established that significantly reduced both the time and technical demands for H3^F^ detection. Meanwhile, the dynamics of H3^F^ throughout the entire cell cycle of *Tetrahymena* were tracked at single-cell resolution. H3^F^ lasted until the post-zygotic stage during conjugation, right before new MAC development, implying its specific role in the MIC and its removal being essential for new MAC formation. H3^F^ displayed a distinct temporal profile from the cell cycle-dependent (mitosis-specific) H3S10ph, supporting the notion that H3 clipping occurs upstream of this modification. All these findings indicated that this antibody-based protocol will serve as a powerful and accessible approach for investigating the regulation and biological significance of H3 clipping in *Tetrahymena*.

## Materials and methods

### *Tetrahymena thermophila* cell culture

Wild-type *Tetrahymena thermophila* strains used in this study were obtained from the *Tetrahymena* Stock Center (https://tetrahymena.vet.cornell.edu). Cells were grown in 1 × SPP medium at 30 ℃ with constant shaking at 120 rpm. Log-phase cultures with a cell density of 2–3 × 10^5^ cells/mL were used for all experiments.

### Immunization of mice

Female BALB/c mice (6–8 weeks old) were purchased from a licensed vendor (Jinan Pengyue Laboratory Animal Breeding Co., Ltd., License No. SCXK [Lu] 20140007). All animal experiments were approved by the animal experimental ethics review committee and carried out in accordance with relevant guidelines.

Mice were acclimated for one week before immunization. Branched peptides synthesized and conjugated to keyhole limpet hemocyanin (KLH) by Shanghai Apeptide Co. (Shanghai, China) served as the antigen for immunization, while non-conjugated peptides were used as coating antigens for enzyme-linked immunosorbent assay (ELISA). Three 2 × branched peptides with different lengths, i.e., (ARKSTGG)_2_-K-C-KLH, namely 9#; (ARKSTGGKA)_2_-K-C-KLH, namely 11#; and (ARKSTGGKAPR)_2_-K-C-KLH, namely 13#, were designed for mice immunization. For each antigen, 3–5 mice were immunized, with additional unimmunized mice included as negative controls.

Primary immunization was performed by intraperitoneal injection of 100 μg KLH-conjugated peptide emulsified in complete Freund’s adjuvant (1:1, v/v). Booster injections were administered using incomplete Freund’s adjuvant at 10-day intervals. Antisera were collected via tail-tip bleeding one week after each immunization to monitor antibody titers by ELISA. Once a stable titer was achieved, a final injection with peptide in PBS (without adjuvant) was performed to enhance the immune response.

### ELISA for antibody titer evaluation

Unconjugated peptides or recombinant histones were coated onto 96-well plates at 50 µg/mL at 4 °C overnight. Plates were then blocked with 5% (w/v) skim milk at 37 °C for 2 h. Diluted mouse antisera (100 µL per well) were added and incubated at 37 °C for 1.5 h, followed by incubation with HRP-conjugated goat anti-mouse IgG as the secondary antibody. TMB substrate was used for color development, and absorbance was measured at 450 nm (OD_450_). The titer was defined as the highest dilution showing an OD_450_ value at about 2.1 times that of the negative control, as the OD_450_ ratio was ≥ 2.1.

### Expression and purification of recombinant H3 proteins

Recombinant expression plasmids for SUMO-H3^F^-6His, HHT1-6His (for full length H3), and other truncated H3 (SUMO-H3 4^5, 5^6, 7^8, 8^9, 9^10, 10^11-6His, “A^B” denotes truncation occurring between the A^th^ and B^th^ amino acids of H3) were constructed in pET22b and transformed into *E. coli* BL21 (DE3). The induction for H3^S^-6His expression was performed using 0.5 mmol/L IPTG at 37 °C for 2–4 h in LB medium. Bacteria were lysed by sonication, and the pellets were washed and solubilized in a 7 mol/L urea-based buffer. The solubilized proteins were further purified by dialysis in a 1 ‰ TFA/DTT solution to remove impurities and then dialyzed into water. SUMO-tagged proteins were purified using Ni–NTA affinity chromatography under denaturing conditions, followed by removal of the SUMO tag through ULP1 cleavage. The proteins were lyophilized and stored at  − 80 °C.

### Histone extraction

MAC and MIC were separated following the protocol previously described (Duan et al. 2021). Briefly, cells were lysed by blending, followed by differential centrifugation to separate MAC and MIC based on size and density. Histones were extracted from the isolated nuclei using the classical acid extraction method. Nuclei pellets were resuspended in 0.2 mol/L sulfuric acid (H_2_SO_4_) and incubated with gentle agitation at 4 °C for several hours to solubilize histones. The acid-soluble fraction was collected by centrifugation at 15,000 rpm for 15 min at 4 °C, and histones were subsequently precipitated by adding trichloroacetic acid (TCA). After washing with acetone, the histone pellet was air-dried and dissolved in ddH_2_O and stored at − 80 °C for downstream analyses.

### Sodium dodecyl sulfate–polyacrylamide gel electrophoresis (SDS–PAGE) and WB

WB samples were prepared according to previously described protocols (Cai et al. 2025). A total of 5 × 10^5^ cells were resuspended in 500 µL of 10% TCA on ice for 30 min. The mixture was then centrifuged at 9000 *g* for 15 min at 4 °C, and the resulting pellet was then resuspended in SDS loading buffer and separated by SDS-PAGE. Proteins were then transferred onto PVDF membranes for Western blot analysis using specific antibodies. The primary antibodies used in this study were: α-H3 (1: 3000, *Tetrahymena*-specific, gift from Dr. Yifan Liu, University of Southern California) and α-H3S10ph (1:5000, *Tetrahymena*-specific, gift from Dr. Yifan Liu), α-H3^F^ (1:1000, generated by this study) and α-tubulin (1:5000, Millipore, CP06). The secondary antibody was Goat anti-Rabbit IgG (H + L) HRP Conjugated (1:8000, TransGen Biotech, HS101) or Goat anti-Mouse IgG (H + L) HRP Conjugated (1:8000, TransGen Biotech, HS201).

### Immunofluorescence (IF) staining

Approximately 5 mL of *Tetrahymena* cells were fixed and permeabilized using 20 μL of fixative solution (saturated HgCl_2_: 95% ethanol = 2:1) at room temperature for 5 min. Cells were washed twice with pre-chilled methanol and resuspended in 1 mL methanol. For single staining, after smearing on glass slides, samples were blocked and incubated with primary antibodies (α-H3^F^, 1:1000; α-H3S10ph, 1:20,000), followed by fluorescently labeled secondary antibodies (1:4000, goat anti-rabbit Alexa Fluor 555 or goat anti-mouse Alexa Fluor 555).

For the co-staining experiment, the fixation procedure was identical to that used for single staining. Samples were first incubated with the H3^F^ primary antibody (1:1000), followed by the corresponding secondary antibody (goat anti-mouse Alexa Fluor 555, 1:4000). After washing, the slides were cross-linked with 3% paraformaldehyde (PFA) for 10 min at room temperature to stabilize the first antibody complex. The samples were then subjected to a second round of staining with the H3S10ph (1:20,000) antibody, followed by its corresponding secondary antibody (goat anti-rabbit Alexa Fluor 488, 1:4000).

## Results

### Generation of H3^F^-specific antibodies

Previous studies have demonstrated that branched peptides can significantly enhance immunogenicity compared to linear peptides (Calvo-Calle et al. 2006; Tam 1988). Based on this rationale, three 2 × branched peptides of different lengths targeting the N-terminal residues specifically exposed in the truncated form of histone H3 (H3^F^) (9#, 11#, and 13#) were designed (Fig. 2B). After obtaining the antibodies, a series of validation assays including ELISA, WB, and IF were performed to assess their specificity and utility, as illustrated in the experimental workflow (Fig. 2C, D).

After the secondary immunization, two mice from each immunized group were randomly selected for blood collection from the tail tip. Antisera were diluted at 1:500 and 1:1000, and ELISA assays were performed to evaluate immune responses against the corresponding antigens. The OD_450_ values were normalized to those from non-immunized mice. The results showed that all groups exhibited immune responses against their respective antigens with ratios ≥ 2.1 (Supplementary Table S1, red ones). After the third immunization, immune responses were again assessed, showing that at least one mouse from each group responded to both H3^F^ and the corresponding peptide with ratios ≥ 2.1 (Supplementary Table S2, red ones). Prior to the final booster immunization, ELISA at 1:500 showed that all three immunized mice exhibited strong immune responses exceeding the 2.1 threshold (9# at 4.39, 11# at 2.55, and 13# at 5.53), confirming the successful generation the successful generation of antibodies with high specificity (Fig. 2E; Supplementary Table S3). Based on ELISA results, working dilutions were determined for each antiserum: 9# and 13# were used at 1:2000 dilution, while 11# was used at 1:1000, with their normalized OD_450_ ratio around 2.1 at these dilutions (Fig. 2E; Supplementary Table S3). These results demonstrated that the branched peptide design effectively elicited immune responses, enabling its subsequent application in WB and IF assays to detect H3^F^.

### High specificity of H3^F^ antibodies

**Fig. 3:**
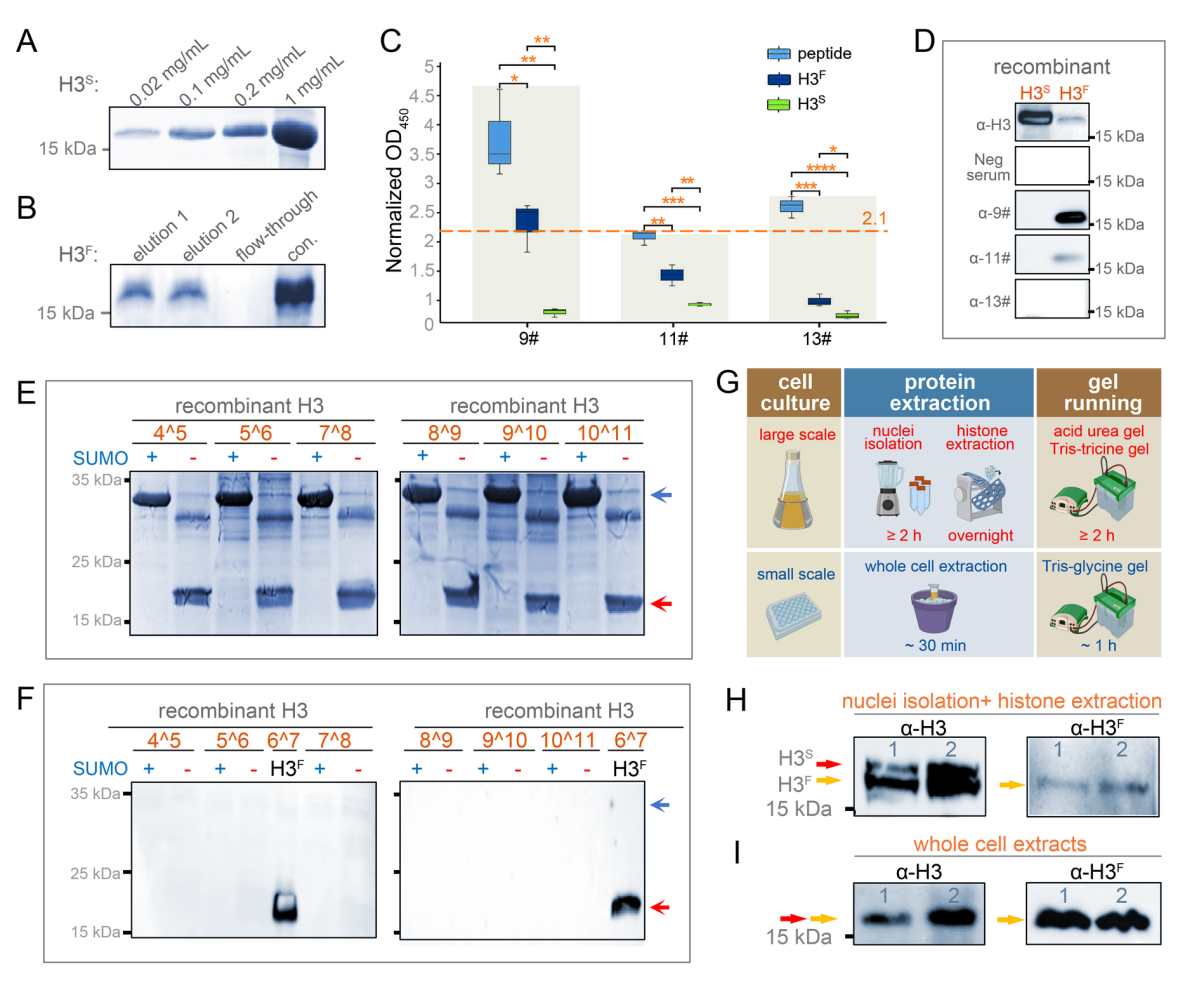
Characterization of H3^F^ antibody in distinguishing H3^F^ from H3^S^. **A** Coomassie blue staining of the recombinant histone H3^S^. Dilution gradients represent 0.02 mg/mL, 0.1 mg/mL, 0.2 mg/mL, and 1 mg/mL. **B** Coomassie blue staining of the recombinant histone H3^F^. Elution 1 and elution 2 are proteins obtained after the ULP1 protease cleavage. Flow-through is filtrate collected from the ultrafiltration tube. Concentrated (con.) is combined elution 1 and elution 2 after concentration. **C** ELISA OD_450_ values of various mice antisera to peptide, H3^F^, and H3^S^, normalized against the control antiserum. All antisera showed immune reactivity to peptides but not to H3^S^. Only 9# exhibited H3^F^ recognition, with the OD_450_ ratio ≥ 2.1 (red dashed line). *P < 0.05; **P < 0.01; ***P < 0.001; ****P < 0.0001. **D** WB of recombinant H3^S^ and H3^F^ using the H3 antibody and different antisera, with anti-serum from non-immunized mice as the negative control. The recombinant proteins were recognized by the H3 antibody but not the negative antiserum. Both 9# and 11# exhibited recognition for H3^F^ but not for H3^S^, with 9# being more effective, while 13# showed no binding ability to either H3^F^ or H3^S^.** E** Coomassie blue staining of recombinant H3 with different truncation lengths. The expressed proteins include SUMO-H3 4^5, 5^6, 7^8, 8^9, 9^10, 10^11-6His, “A^B” denotes truncation occurring between the A^th^ and B^th^ amino acids of H3. The blue “ + ” symbol/arrow refers to the SUMO-H3 A^B-6His, while the red “-” symbol/arrow indicates the proteins after SUMO removal.** F** WB result using H3^F^ antibody for different length truncated H3 detection. The H3^F^ antibody recognized only H3^F^ truncated after the first six amino acid. The blue “+” symbol/arrow refers to proteins with the SUMO tag, while the red “-” symbol/arrow indicates proteins after the SUMO tag removal.** G** Comparison between traditional (top panel) and H3^F^ antibody-based (down panel) workflows for H3^F^ detection. Traditional detection required large-scale culture, nuclear isolation (≥ 2 h), overnight histone extraction, and gel-based separation (acid urea or Tris-tricine gel, ≥ 2 h), followed by WB using H3 antibody. The H3^F^ antibody enabled direct detection from small-scale cultures (1 mL), through TCA precipitation (30 min), tris–glycine SDS-PAGE (1 h), and WB.** H** WB detection for in vivo H3^F^ testing by traditional nuclei isolation and histone extraction. The left panel employed the H3 antibody, while the right panel used the H3^F^ antibody by stripping the H3 antibody from the same PVDF membrane. Lanes 1 and 2 indicate the duplicated samples. The red and orange arrows refer to H3^S^ and H3^F^, respectively.** I** WB detection for in vivo H3^F^ testing using whole cell extraction. The left panel shows the result obtained with the H3 antibody, while the right panel shows that of the H3^F^ antibody. Lanes 1 and 2 indicate duplicated samples. The red and orange arrows refer to H3^S^ and H3^F^, respectively

To evaluate the specificity of the antisera, recombinant H3 proteins were expressed and purified in *E. coli*. The full-length H3 (H3^S^) was expressed as inclusion bodies, which were solubilized and purified under denaturing conditions. Meanwhile, the truncated H3 (H3^F^) was expressed in a soluble form and purified using Ni–NTA affinity chromatography. Both recombinant proteins showed high yield and purity in Coomassie-stained SDS-PAGE gels (Fig. 3A, B). WB was performed with an H3 antibody, which recognized both H3^F^ and H3^S^, validating the recombinant products (Fig. 3D, top panel).

Next, ELISA assays were performed to assess the binding specificity of antisera to recombinant H3^F^ and H3^S^, as well as synthetic peptides, using the working concentration determined before. As expected, all three antisera exhibited strong responses to the immunizing peptides, with ELISA titers well above the 2.1-fold threshold compared to negative control antiserum, i.e., 3.8 for 9#, 2.1 for 11#, and 2.6 for 13# (Fig. 3C; Supplementary Table S4). However, differential reactivity was observed when full-length and truncated H3 were tested. Only antiserum from mouse 9# showed a strong signal against H3^F^ (OD_450_ ratio = 2.3), while 11# showed weak reactivity (ratio = 1.4) and 13# had almost no response (ratio = 1) (Fig. 3C; Supplementary Table S4). Importantly, none of the antisera exhibited any detectable binding to H3^S^ (Fig. 3C; Supplementary Table S4), indicating minimal cross-reactivity with the full-length protein. To confirm this observation, WB analysis was performed using the same antisera against the recombinant H3^F^ and H3^S^ proteins. Consistent with the ELISA result, antiserum from mouse 9# specifically and strongly recognized H3^F^, 11# showed faint signals, and 13# showed no reactivity. All three antisera failed to detect H3^S^, reaffirming their specificity for H3^F^ (Fig. 3D). Thus, antiserum 9# was selected for subsequent experiments as the H3^F^-specific antibody.

To rigorously test the epitope specificity, several additional truncated variants of H3 were cloned and expressed, each lacking different length of N-terminal amino acids of H3. Specifically, truncated variants starting from the 5^th^, 6^th^, 8^th^, 9^th^, 10^th^, and 11^th^ amino acids of H3 were purified and their recognition by the antisera was evaluated via WB (Fig. 3E, F). The H3^F^ antibody exclusively recognized the correctly truncated form H3^F^, but not the other truncated variants (Fig. 3F). This result strongly supports the conclusion that the antibody is both specific and sensitive to the precise cleavage event generating H3^F^, making it a reliable tool for studying H3 clipping in *Tetrahymena*.

### Simplified detection of H3^F^ in *Tetrahymena*

Traditionally, detection of H3^F^ in *Tetrahymena* required the isolation of MICs from large scale cultures (with at least 1 × 10^8^ cells) followed by acid extraction of histones, a labor-intensive and time-consuming process. Extracted histones are then resolved using two-dimensional (2D) acid/urea gel or tris-tricine gel (Allis et al. 1980, 1979; Allis and Wiggins 1984b; Papazyan et al. 2014). 2D-AU gels separate histones based on charge, modifications, and effective molecular size, and Tris-Tricine SDS-PAGE is employed for its superior ability to resolve low-molecular-weight proteins (below 20 kDa), which is essential for effectively separating the full-length H3^S^ and the 6-amino-acid-truncated H3^F^. Both electrophoresis methods need more than two hours to achieve sufficient separation between H3^S^ and H3^F^, and then WB is performed using the pan-H3 antibody to reveal the two distinct bands of H3 in MIC (Fig. 3G, top panel). To evaluate the specificity of the newly generated H3^F^ antibody, H3^S^ and H3^F^ were separated as described above and WB was performed using the H3 antibody, which revealed two distinct bands (Fig. 3H, left). After antibody stripping, the membrane was reintubated with the H3^F^-specific antibody, which exclusively recognized the lower band corresponding to H3^F^ (Fig. 3H, right). This experiment confirmed that the H3^F^ antibody specifically detected truncated H3 in *Tetrahymena* histone extracts.

To streamline H3^F^ detection, whole-cell extract (WCE) was tested as a substitute for conventional histone preparations. *Tetrahymena thermophila* cells were cultured in small volumes (2 × 10^5^ cells in 1 mL), and lysed proteins were separated by standard tris–glycine SDS-PAGE (~ 1 h) (Fig. 3G, lower panel). Remarkably, the H3^F^ antibody successfully detected the H3^F^ band in WCE samples (Fig. 3I, right panel). In contrast, the H3 antibody failed to resolve two separated bands (Fig. 3I, left panel), demonstrating the specificity and robustness of the H3^F^ antibody for detecting H3^F^ in small-scale, simplified conditions.

Taken together, this newly developed antibody significantly reduces the time, labor, and input requirement for H3^F^ detection, thus facilitating rapid and scalable investigation of H3^F^ dynamics in various cellular conditions.

### H3 clipping is an upstream event of H3S10 phosphorylation

**Fig. 4:**
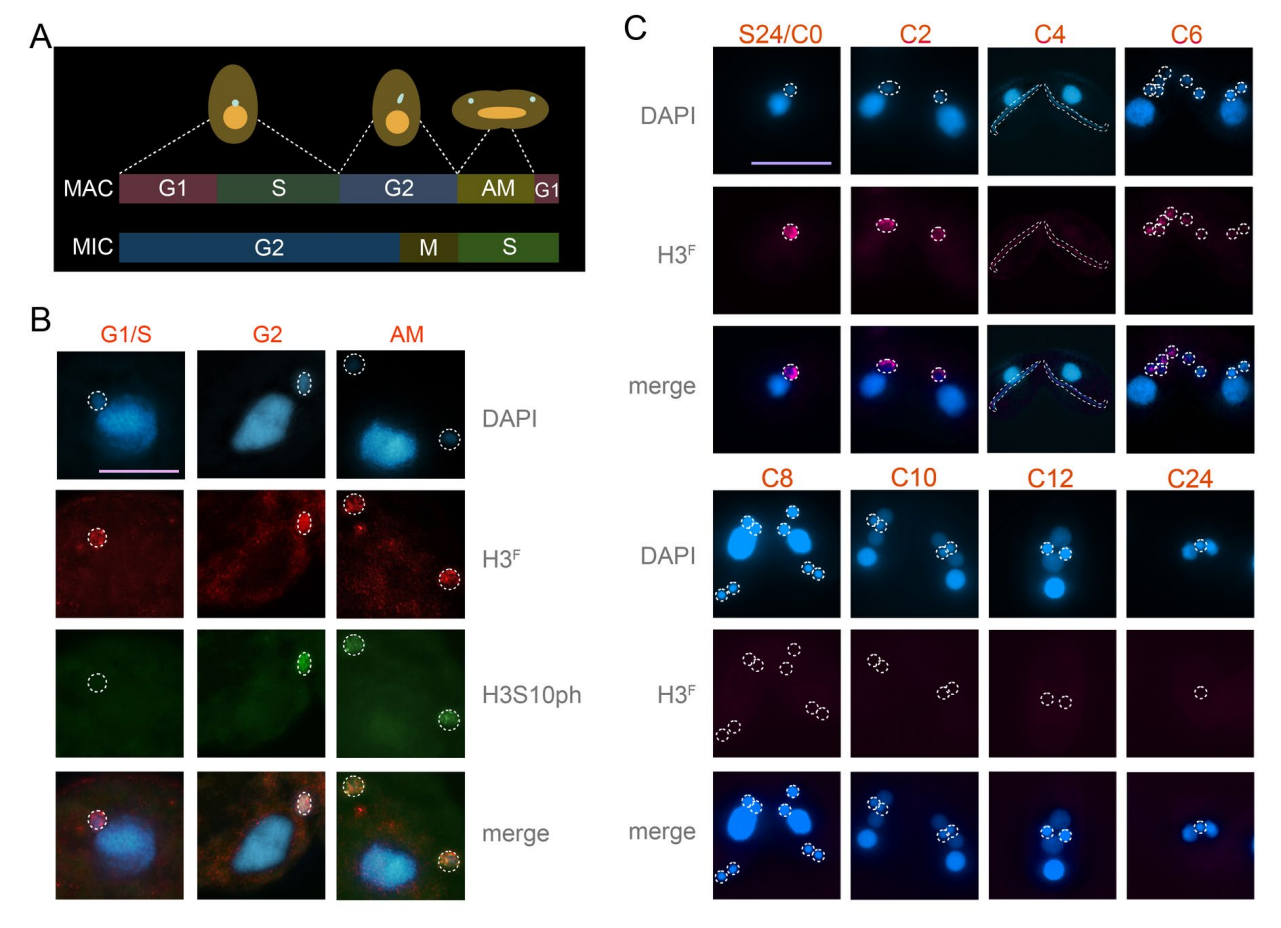
H3^F^ dynamics during different cell stages. **A** Diagram of the cell cycle of *Tetrahymena*. The top panel represents MAC cycles; the lower panel depicts MIC cycles. The germline MIC divides by mitosis without G1 phase, whereas the somatic MAC divides by amitosis (AM). **B** H3^F^ and H3S10ph localization during the vegetative stage. H3^F^ was consistently detected in the MIC throughout the vegetative cell cycle. H3S10ph signals appeared specifically during G2 and AM phases. G1, gap 1 phase; S, synthesis phase; G2, gap 2 phase; AM, amitosis. The dashed outlines indicate the MIC. Scale bar, 5 μm.** C** H3^F^ localization during starvation and conjugation. C0, 2, 4, 6, 8, 10, 12, and 24 refers to 0, 2, 4, 6, 8, 10, 12, and 24 h after the initiation of conjugation. H3^F^ was present in cells starved for 24 h (S24), and during early conjugation until C6. The dashed outlines indicate the MIC. Scale bar, 20 μm

To compare the distribution pattern and establish the upstream–downstream relationship of H3^F^ with H3S10ph, the H3^F^ and H3S10ph antibodies were used for IF co-staining. It should be noted that in *Tetrahymena*, the somatic MAC undergoes an amitotic division cycle comprising the G1, S, G2, and AM (amitosis) phases, which parallels the canonical G1-S-G2-M pattern of mitotic division in other eukaryotes (Cole and Sugai 2012). In contrast, the germline MIC divides by conventional mitosis but lacks a distinct G1 interval, and its S phase and mitotic events are temporally uncoupled from those of the MAC (Fig. 4A) (Liu et al. 2021). Given the asynchronous nature of these two nuclear cycles, all stages described in our IF analyses were defined according to the corresponding phase of the MAC cycle to ensure consistency and facilitate direct comparison across samples. IF result showed that the H3^F^ antibody produced clear and MIC- specific signals throughout the entire vegetative cell cycle (Fig. 4B). In contrast, H3S10ph exhibited phase-specific signals, only appearing in G2 and AM stages (Fig. 4B), consistent with previous reports (Wei et al. 1998). Taking into account that H3^F^ remains detectable in H3S10A mutants (Wei et al. 1999), the temporal restriction of H3S10ph, despite the continuous presence of H3^F^, provides strong evidence that H3S10ph is a downstream event following H3 clipping, rather than a prerequisite or initiating signal. It also indicates that H3S10ph does not fully reflect the temporal profile of H3^F^, therefore it is not appropriate to serve as an alternative indicator of H3^F^.

### H3^F^ disappeared before the new MAC formation

To further investigate the temporal presence of H3^F^ in *Tetrahymena* cells during different cell stages, IF staining was performed using the newly generated H3^F^ antibody during starvation and conjugation. H3^F^ signal persisted in the MIC after the 24 h starvation (S24) (Fig. 4C), which also served as the time point for mixing cells to initiate mating, designated as conjugation 0 (C0). H3^F^ remained detectable during the early conjugation stages, persisting up to six hours post-conjugation (C6), a phase during which nuclear exchange was occurring (Fig. 4C). However, after nuclear exchange at approximately eight hours post-conjugation (C8), when the zygotic nucleus is selected and initiates differentiation into the new MIC and the new MAC, H3^F^ signal rapidly diminished and became undetectable thereafter (Fig. 4C). This finding provided direct evidence supporting the hypothesis that the disappearance of H3 clipping is a prerequisite for new MAC development, offering new insights into the temporal regulation of H3 clipping during nuclear differentiation.

## Discussion

In this study, a highly specific antibody against the truncated form of histone H3 (H3^F^) in *Tetrahymena thermophila* was successfully developed and validated. By employing a rational epitope-focused strategy (Calvo-Calle et al. 2006), 2 × branched peptides were designed mimicking the N-terminal region of H3^F^, which elicited strong immunogenicity while maintaining high epitope specificity. Specificity was rigorously validated by ELISA, WB, and IF assays using synthetic peptides, recombinant H3 proteins with varying N-terminal truncations, and in situ samples. The development of this H3^F^-specific antibody provides a direct readout of H3 clipping and eliminates the need for labor-intensive methods (Allis et al. 1979; Papazyan et al. 2014; Smith et al. 2008). Using this antibody, we resolved temporal patterns of H3^F^ that are distinct from H3S10ph (Wei et al. 1998, 1999), placing H3S10ph downstream of H3 clipping. This study also represents the first comprehensive tracking of H3^F^ dynamics across different cellular stages, revealing its presence during vegetative growth, starvation, and early conjugation, and linking the disappearance of H3^F^ with new MAC development.

**Fig. 5:**
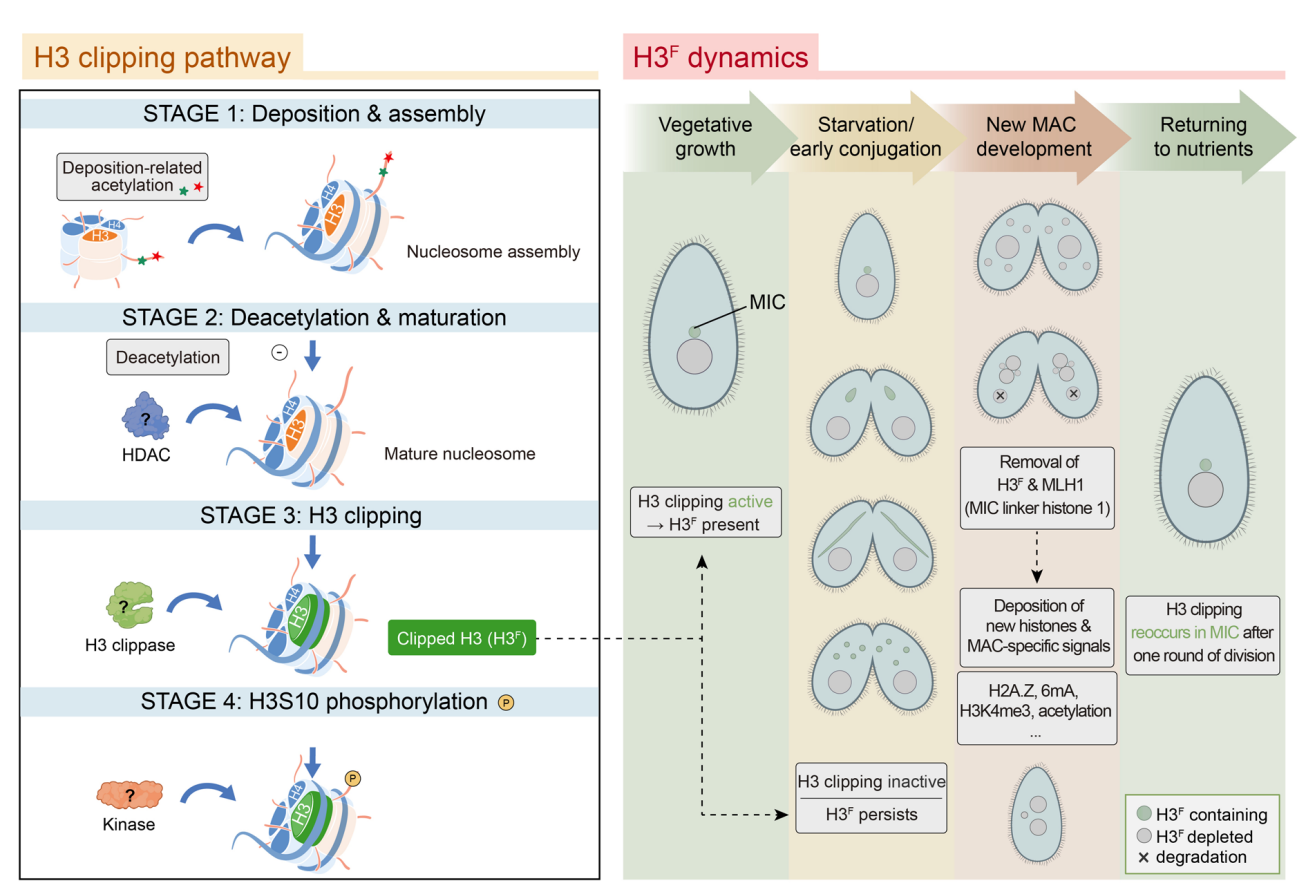
A proposed model of the histone H3 clipping pathway and summarization of H3^F^ dynamics across the *Tetrahymena* life cycle. As shown in the left panel, synthesizing our findings with previously reported data, we proposed a four-stage sequential model for H3 proteolytic processing: Stage 1, where newly synthesized H3 undergoes deposition-related acetylation for nucleosome assembly; Stage 2, in which mature chromatin is deacetylated by histone deacetylase (HDAC) to prepare the substrate; Stage 3, where the unknown H3 clippase acts at the nucleosomal level to generate the truncated H3^F^; and Stage 4, where phosphorylation at the serine 10 residue is catalyzed by an unknown kinase. The right panel delineates the spatiotemporal dynamics of H3^F^, showing that H3 clipping is active during vegetative growth to produce H3^F^. During starvation and early conjugation, although new clipping ceases, H3^F^ persists, suggesting its role in maintaining chromatin stability. In the new MAC development stage, H3^F^ is actively removed along with micronucleus-specific markers like MLH1, likely through a replacement mechanism driven by incorporation of new histones. This transition is marked by the establishment of transcriptionally active macronuclear epigenetic signatures, including DNA N^6^-adenine methylation (6mA), H3K4me3, histone acetylation, and H2A.Z. Finally, upon returning to nutrients and undergoing one round of cell division, H3 clipping re-emerges specifically within the MIC

It has previously been reported that the deposition-associated histone deacetylation is indispensable for *Tetrahymena* H3 clipping, implying that clipping occurs after nucleosome assembly (Smith et al. 2008). Here we confirmed that H3S10ph follows H3 clipping. Integrating these findings with the histone deposition pathway (Allis et al. 1985; Smith et al. 2008), we outlined the sequence of events as follows (Fig. 5, left panel): deposition-related acetylation facilitates nucleosome assembly; subsequent deacetylation marks chromatin maturation; H3 clipping occurs on nucleosomal histones; and finally, H3S10ph is established. This cascade highlights that H3 clipping is not an isolated event but part of a coordinated process, providing a framework for understanding how histone proteolytic processing integrates into epigenetic regulation to shape chromatin function.

Previous studies showed that H3 clipping is restricted to vegetative growth but absent during starvation or conjugation (Allis and Wiggins 1984b; Allis et al. 1980; Papazyan et al. 2014). Our IF analysis revealed that the clipped form H3^F^ persisted through starvation and remained detectable in early conjugation. This persistence suggests a potential role for H3^F^ in meiosis or pronuclear selection, in line with the observation that H3K23me3, a modification contributing to DNA protection during meiosis, occurs exclusively on H3^F^ at early conjugation (Papazyan et al. 2014). During the transition from the zygotic MIC to the new MAC, H3^F^, along with the MIC-specific linker histone MLH1, are eliminated (Fig. 5, right panel) (Allis and Wiggins 1984a, b; Nabeel-Shah et al. 2020). The abrupt disappearance of H3^F^ around the new MAC development stage suggests an active removal or replacement process, likely through large-scale incorporation of newly synthesized histones (Allis and Wiggins 1984a; Chalker 2008; Liu et al. 2004). Such turnover is accompanied by the establishment of MAC-specific transcription-associated PTMs, such as DNA N^6^-methyladenine (6 mA), H3K4me3, histone acetylation, and the histone variant H2A.Z (Fig. 5, right panel) (Allis and Wiggins 1984a; Cheng et al. 2025; Cui et al. 2006; Wahab et al. 2020). The reoccurrence of H3 clipping in the MIC of progeny cells is only detected when cells return to nutrient-rich medium and complete one round of vegetative division (Fig. 5, right panel) (Allis and Wiggins 1984b). Collectively, these findings underscore that monitoring H3^F^ dynamics provides a powerful tool for dissecting the specific functions of H3^F^ in nuclear development, chromatin remodeling, and the cell cycle.

Although compelling evidence for H3 clipping has been obtained in *Tetrahymena*, the protease(s) responsible for generating H3^F^ remain unidentified (Wei et al. 2022). Proteases are highly abundant in *Tetrahymena*, accounting for approximately at least 25% of the total proteome (Madinger et al. 2010). Yet their substrate specificities are largely uncharacterized, and potential overlap in activities further complicates the identification of the clipping protease(s) (Orhan et al. 2024). We attempted to narrow down potential candidates through homology-based searches for cathepsin B-, C-, L-like and glutamate dehydrogenase family cysteine proteins (Wei et al. 2022), but thus far identified no candidates with clear MIC localization. To address this, we are pursuing complementary strategies, including inhibitor-based assays and site-directed mutagenesis around the cleavage site, to probe the biochemical properties and substrate preferences of the responsible protease(s). In parallel, we are adapting TurboID for *Tetrahymena* to generate a comprehensive and high-confidence MIC proteome, which will serve as a valuable reference for systematic search efforts. Another unresolved question is how H3 clipping is modulated by epigenetic contexts and whether the exclusive presence of H3^F^ in the MIC results from protease restriction to the MIC or from inhibitions in the MAC. This H3^F^-specific antibody enables direct and sensitive detection of clipping, offering a powerful tool to identify the designated protease(s), elucidate their regulation, and define the cellular functions of H3^F^.

Most studies of H3 clipping rely heavily on in vitro assays (Azad and Tomar 2016; Mahendra and Kanungo 2000; Roquis et al. 2021; Sandoval-Basilio et al. 2016; Santos-Rosa et al. 2009). Although informative, these assays may be affected by artifacts from sample preparation, including processing-induced proteolytic artifacts or degradation (Azad et al. 2018; Dhaenens et al. 2015; Tilley et al. 2022), making it difficult to fully establish H3 clipping as a *bona fide* intracellular event. For example, biotinylated histone H3 has been shown to undergo proteolytic degradation even under controlled conditions, raising concerns about the reliability of in vitro observations (Vossaert et al. 2014). By contrast, in mouse embryonic stem cells, cathepsin L-mediated H3 clipping was characterized in vivo using an antibody specific to the cleaved product (between Ala21 and Thr22), which enabled identification of the responsible protease cathepsin L, its mechanism, functional outcomes, as well as structural basis of how cathepsin L engages H3 (Adams-Cioaba et al. 2011; Duncan et al. 2008). Therefore, subcellular localization-based approaches, like the antibody-based in situ detection method developed here, are essential for characterizing H3 clipping as a physiologically relevant process.

## Supplementary Information

Below is the link to the electronic supplementary material.

Supplementary file1 (DOCX 28 KB)

## Data Availability

Datasets generated or analyzed during this study are included in this article and its supplementary material. The raw data supporting the conclusions of this article will be made available by the authors, without undue reservation.
