# Supplementary material for: Specific tracking of N-terminal clipping on histone H3 in Tetrahymena enabled by a custom branched-peptide antibody
Source: Mar Life Sci Technol. 2026 Jan 14;8(2):324–36. doi: 10.1007/s42995-025-00351-4 (PMC13198579; doi:10.1007/s42995-025-00351-4)
Supplement: Supplementary file 1 — Supplementary file1 (DOCX 28 KB) [file 42995_2025_351_MOESM1_ESM.docx]

**Supplemental materials**

**Table S1. Normalized OD_450_ values (duplicated) against the corresponding antigen peptides after the secondary immunization.**

| **Antisera** | **Antigen** | **Mouse 1** | | **Mouse 2** | |
| --- | --- | --- | --- | --- | --- |
|  |  | **1:500** | **1:1000** | **1:500** | **1:1000** |
| **9#** | **9# peptide** | 5.77^*^ | 3.44^*^ | 4.78^*^ | 2.46^*^ |
| **11#** | **11# peptide** | 9.58^*^ | 6.11^*^ | 9.20^*^ | 5.37^*^ |
| **13#** | **13# peptide** | 11.66^*^ | 6.81^*^ | 9.13^*^ | 6.14^*^ |

^*^ Normalized OD_450_ values ≥ 2.1 are highlighted in red.

| **Antisera** | **Antigen** | **Mouse 1** | | **Mouse 2** | |
| --- | --- | --- | --- | --- | --- |
|  |  | **1:500** | **1:1000** | **1:500** | **1:1000** |
| **9#** | **H3^F^** | 3.11^*^ | 1.77 | 1.84 | 1.33 |
|  | **9# peptide** | 3.98^*^ | 1.84 | 2.68^*^ | 1.92 |
| **11#** | **H3^F^** | 2.19^*^ | 1.57 | 2.12^*^ | 1.67 |
|  | **11# peptide** | 3.01^*^ | 2.20^*^ | 2.61^*^ | 2.09 |
| **13#** | **H3^F^** | 2.11^*^ | 1.33 | 1.79 | 1.09 |
|  | **13# peptide** | 3.96^*^ | 2.34^*^ | 1.50 | 0.80 |

**Table S2. Normalized OD_450_ values (triplicated) against the corresponding antigen peptides and recombinant H3^F^ after the third immunization.**

^*^ Normalized OD_450_ values ≥ 2.1 are highlighted in red.

**Table S3. Normalized OD_450_ values (triplicated) at different antisera dilution ratios after the fourth immunization.**

|  | **1: 500** | **1: 1000** | **1: 2000** | **1: 4000** | **1: 8000** | **1: 16,000** | **1: 32,000** |
| --- | --- | --- | --- | --- | --- | --- | --- |
| **9#** | 4.39^*^ | 2.85^*^ | 1.80 | 0.94 | 0.55 | 0.35 | 0.24 |
| **11#** | 2.55^*^ | 1.73 | 0.66 | 0.45 | 0.19 | 0.17 | 0.16 |
| **13#** | 5.53^*^ | 3.75^*^ | 2.42^*^ | 1.30 | 0.62 | 0.46 | 0.38 |

^*^ Normalized OD_450_ values ≥ 2.1 are highlighted in red.

**Table S4. Normalized OD_450_ values (triplicated) of various mice antisera to peptide, H3^F^, and H3^S^ after the final immunization.**

|  | **peptide** | **H3^F^** | **H3^S^** |
| --- | --- | --- | --- |
| **9#** | 3.8^*^ | 2.3^*^ | 0.8 |
| **11#** | 2.1^*^ | 1.4 | 0.9 |
| **13#** | 2.6^*^ | 1 | 0.7 |

^*^ Normalized OD_450_ values ≥ 2.1 are highlighted in red.
